# Extracellular Vesicles from Bronchoalveolar Lavage Fluid Indicate Early Biomarker Potential and Differentiate Local Lung Injury in a Porcine Model of Asymmetric Acute Lung Injury

**DOI:** 10.3390/ijms27167173

**Published:** 2026-08-11

**Authors:** Benjamin Seybold, Anna Lena Jung, Lynn Feuerbach, Thomas Heimerl, Claudine H. Mutschler, Nils Englert, Cleo-Aron Weis, Tanja Poth, Markus A. Weigand, Armin Kalenka, Mascha O. Fiedler-Kalenka

**Affiliations:** 1 Department of Anesthesiology, Heidelberg University Hospital, Medical Faculty, University Heidelberg, 69120 Heidelberg, Germanymascha.fiedler-kalenka@med.uni-heidelberg.de (M.O.F.-K.); 2 Institute for Lung Research, Universities of Giesen and Marburg Lung Center, German Center for Lung Research (DZL), Philipps-University Marburg, 35043 Marburg, Germany; anna.jung@uni-marburg.de (A.L.J.);; 3 Core Facility Flow Cytometry–Bacterial Vesicles, Philipps-University Marburg, 35043 Marburg, Germany; 4 Center for Synthetic Microbiology (SYNMIKRO), Philipps-University Marburg, 35043 Marburg, Germany; 5 Translational Lung Research Center Heidelberg (TLRC), German Center for Lung Research (DZL), University Heidelberg, 69120 Heidelberg, Germany; 6 Institute of Pathology, Heidelberg University Hospital, Medical Faculty, University Heidelberg, 69120 Heidelberg, Germany; cleo-aron.weis@med.uni-heidelberg.de; 7 Center for Model System and Comparative Pathology (CMCP), Heidelberg University Hospital, 69120 Heidelberg, Germany; 8 District Hospital Bergstrasse, 69646 Heppenheim, Germany

**Keywords:** acute lung injury, acute respiratory distress syndrome, extracellular vesicles, bronchoalveolar lavage fluid, biomarker, porcine model

## Abstract

Early detection of acute lung injury (ALI) remains challenging, as conventional diagnostics rarely capture initial molecular changes. We therefore examined whether extracellular vesicles (EVs) in bronchoalveolar lavage fluid (BALF) can detect early regional injury and distinguish initial stress mechanisms in a porcine ALI model. Unilateral ALI was induced using Triton X-100, followed by six hours of mechanical ventilation with either fixed positive end-expiratory pressure (PEEP) at 5 cmH_2_O or transpulmonary-pressure (TPP)-guided PEEP. Spatially separated BALF sampling allowed direct comparison between injured and contralateral mechanically stressed lungs. EV concentration and size distribution were quantified by nano-flow cytometry, while vesicular identity was confirmed by transmission electron microscopy and tetraspanin dot blot analyses. Furthermore, EV metrics were related to corresponding histologic injury scores. Across both PEEP strategies, EV concentration was markedly higher in both directly injured and contralateral mechanically stressed lungs compared with sham controls, indicating alveolar stress beyond the primary injury site. Median EV size differed significantly by injury type and correlated inversely with histologic injury, particularly with alveolar neutrophil infiltration. BALF-derived EV concentration and size may therefore provide complementary, spatially resolved molecular readouts of early ALI.

## 1. Introduction

Extracellular vesicles (EVs) are lipid bilayer-enclosed nanoparticles released by virtually all cell types and mediate intercellular communication by reflecting the functional state of their parent cells [1]. In line with current International Society for Extracellular Vesicles recommendations, the term “extracellular vesicles” is used as a generic descriptor without assumptions regarding biogenesis [2]. In this study, EVs denote extracellular vesicular particles <200 nm in diameter detected in bronchoalveolar lavage fluid (BALF) from porcine lungs, corresponding to the MISEV2023 operational category of small EVs.

Acute lung injury (ALI) and its clinical correlate, acute respiratory distress syndrome (ARDS), are characterized by inflammatory disruption of the alveolar–capillary barrier, leading to alveolar edema, impaired gas exchange, and high morbidity and mortality [3]. By definition, ARDS becomes clinically apparent only in advanced stages of lung injury [4]. Invasive mechanical ventilation remains essential in acute respiratory failure but entails a risk of ventilator-induced lung injury (VILI), underscoring the need for early biomarkers to identify vulnerable lung systems before overt ARDS develops [5]. Importantly, ARDS frequently evolves from initially asymmetric lung injury, defining an early ALI phase in which non-injured regions may still be protected [6]. During this phase, individualized ventilator settings—particularly positive end-expiratory pressure (PEEP) titration—are critical, although optimal levels remain poorly defined at the molecular level [7].

By conveying molecular signatures of cellular activation, stress, or injury, EVs have emerged as promising biomarkers across pathologic conditions [8]. In ALI, EV-mediated crosstalk between immune and structural lung cells contributes to pulmonary inflammation [9,10]. Accordingly, evaluating EVs as biomarkers requires contextualization with established molecular and histopathologic indicators of ALI.

So far, research in ARDS has focused primarily on systemically circulating EVs, whereas data on BALF-derived EVs reflecting the site of inflammation remain scarce [8,11]. Profiling BALF-derived EVs may therefore provide mechanistic insight and support early detection of pulmonary stress and ALI, as well as the evaluation of ventilation strategies. To address this gap, we established a translational porcine model of unilateral ALI allowing controlled analysis of EV release from mechanically stressed and inflamed lung regions in relation to tissue pathology.

We investigated whether quantitative and size-based profiling of BALF-derived EVs identifies early molecular alterations in unilateral ALI and correlates with characteristic histologic injury patterns. We further examined whether lung-protective PEEP strategies—fixed low PEEP versus individualized PEEP targeting a slightly positive transpulmonary pressure at end-expiration (TPP_exp_)—result in distinct EV profiles.

## 2. Results

All lavaged animals (n = 17) developed unilateral ALI, as confirmed by previously reported physiologic, computed tomographic, and histologic findings [12]. ALI induction resulted in severe physiologic deterioration, including marked impairment of oxygenation and reduced respiratory system compliance, accompanied by pronounced unilateral loss of aeration on inspiratory and expiratory computed tomography, predominantly confined to the lavaged lung. Histologic injury was likewise highly asymmetric, with higher lung injury scores (LIS) in directly injured lungs than in contralateral and sham lungs. Specifically, directly injured lungs exhibited extensive intra-alveolar edema, septal thickening, and marked alveolar neutrophil infiltration, whereas contralateral lungs showed only mild interstitial inflammatory changes with preserved alveolar architecture, comparable to sham animals [12]. Previous analyses of this lung injury model further demonstrated a redistribution of tidal volume toward the non-lavaged lung during mechanical ventilation, resulting in increased regional mechanical stress despite only minor histologic alterations in these lungs after six hours [13]. Together, these findings established a reproducible model of early asymmetric ALI characterized by direct inflammatory injury in the lavaged lung and secondary mechanical stress in the initially non-injured contralateral lung.

Of these 17 animals, three developed a pneumothorax following ALI induction, which was detected by ultrasound. Because pneumothorax can substantially alter respiratory mechanics and ventilation distribution, these animals were excluded from further analysis. Consequently, the final study population comprised 14 animals: six ventilated with a fixed PEEP of 5 cmH_2_O (ALI_PEEP5; n = 6), six ventilated with an individualized PEEP strategy targeting a TPP_exp_ of 0–3 cmH_2_O (ALI_TPP; n = 6), and two sham animals also ventilated with a fixed PEEP of 5 cmH_2_O but without ALI induction (sham; n = 2). Because BALF and histologic samples were collected separately from the left and right lungs, each lung was analyzed as an individual biological sample. In sham animals, no distinction between directly and indirectly injured lungs was applicable; therefore, all four lungs served as non-injured control samples.

### 2.1. EV Findings from BALF

BALF serves as a direct indicator of alveolar conditions and allows characterization of EV profiles reflecting the site of injury [14]. Samples were obtained from both left and right lungs separately after six hours of mechanical ventilation, prior to euthanasia.

#### 2.1.1. Characterization of BALF-Derived EVs

**Figure 1:**
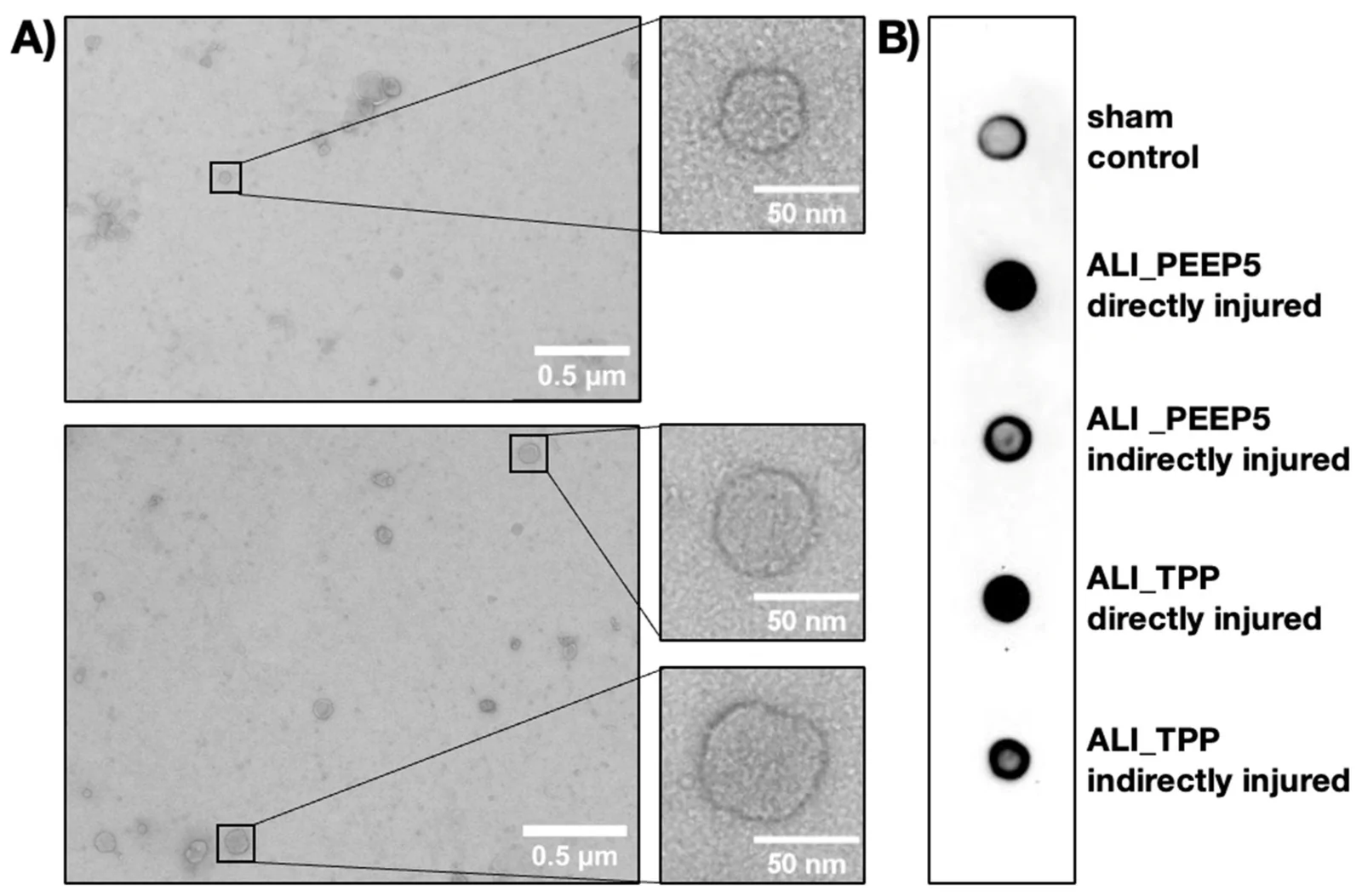
**Morphological and molecular characterization of BALF-derived EVs.** (**A**) Representative transmission electron microscopy (TEM) images of extracellular vesicles (EVs) isolated from bronchoalveolar lavage fluid (BALF). Low-magnification images provide an overview of the isolated BALF-derived vesicle preparations, whereas higher-magnification images demonstrate the characteristic spherical morphology and intact membrane structure of individual EVs. (**B**) Representative Dot blot analysis of isolated BALF-derived vesicle preparations using a tetraspanin antibody cocktail (CD9, CD63, and CD81). Detection of canonical EV-associated tetraspanins supports the vesicular identity of the isolated particles.

To confirm the vesicular identity of extracellular particles in BALF, representative BALF-derived EV preparations were characterized using complementary transmission electron microscopy (TEM) and dot blot analyses. TEM demonstrated abundant membrane-enclosed vesicles with the characteristic spherical morphology and intact membrane structure expected for EVs (Figure 1A). Dot blot analysis further confirmed the presence of the canonical EV-associated tetraspanins CD9, CD63, and CD81 in all analyzed BALF-derived particle preparations (Figure 1B), supporting the vesicular identity of the detected particles.

#### 2.1.2. EV Concentration in BALF

**Figure 2:**
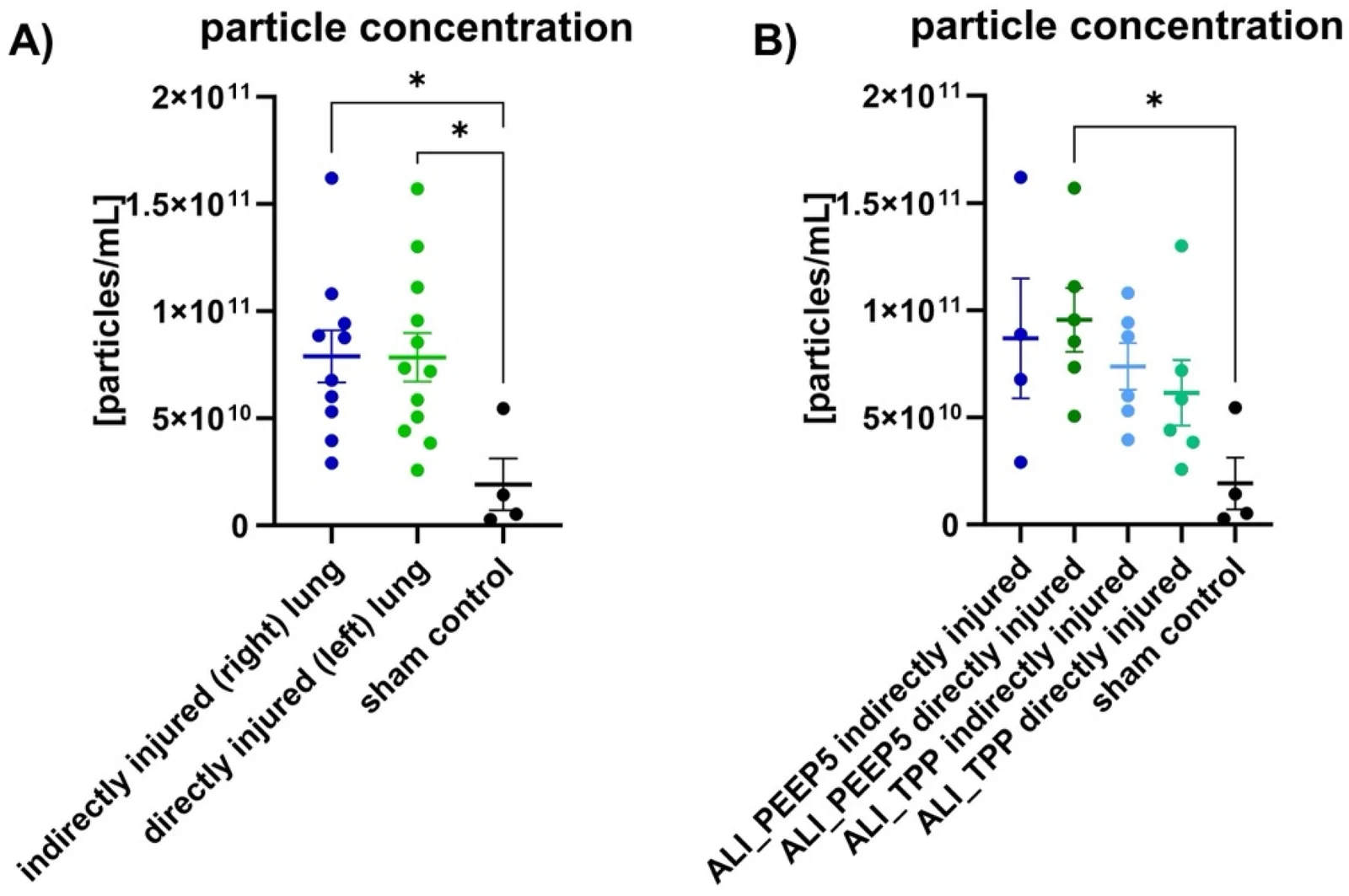
**BALF-derived EV concentrations.** (**A**) Particle concentrations of extracellular vesicles (EVs) measured in bronchoalveolar lavage fluid (BALF) obtained from the indirectly injured (right) lung, the directly injured (left) lung, and sham controls in the porcine acute lung injury model. (**B**) EV particle concentrations from the same lungs, comparing fixed positive end-expiratory pressure (PEEP; 5 cmH_2_O) with individualized PEEP targeting a transpulmonary pressure at end-expiration of 0–3 cmH_2_O. EV concentrations were quantified by nano-flow cytometry (NanoAnalyzer) and samples were analyzed in a blinded manner. Data represent biological replicates from individual lungs, displayed as individual data points with line at mean ± SEM. Sample numbers may differ because predefined outliers were excluded. Statistical analysis was performed using one-way ANOVA with Tukey’s post hoc test; significance is indicated by asterisks (*). Blue = indirectly injured lung; green = directly injured ALI lung; black = sham.

EV concentration in BALF was measured by nano-flow cytometry (NanoFCM) and first analyzed independent of ventilation strategy. Mean EV concentration differed significantly among directly injured, indirectly injured, and sham lungs (*p* = 0.0260), with higher concentrations in both directly injured (7.85 ± 1.14 × 10^10^ particles/mL) and contralateral indirectly injured lungs (7.90 ± 1.22 × 10^10^ particles/mL) compared with sham lungs (1.92 ± 1.20 × 10^10^ particles/mL). No difference was observed between directly and indirectly injured lungs (Figure 2A).

When stratified by ventilation strategy, TPP-guided PEEP was associated with lower EV concentrations than fixed low PEEP, most pronounced in directly injured lungs (6.14 ± 1.52 × 10^10^ vs. 9.55 ± 1.49 × 10^10^ particles/mL). Given the limited subgroup sizes, this difference did not reach statistical significance (*p* = 0.5090; Figure 2B).

#### 2.1.3. EV Size Distribution in BALF

**Figure 3:**
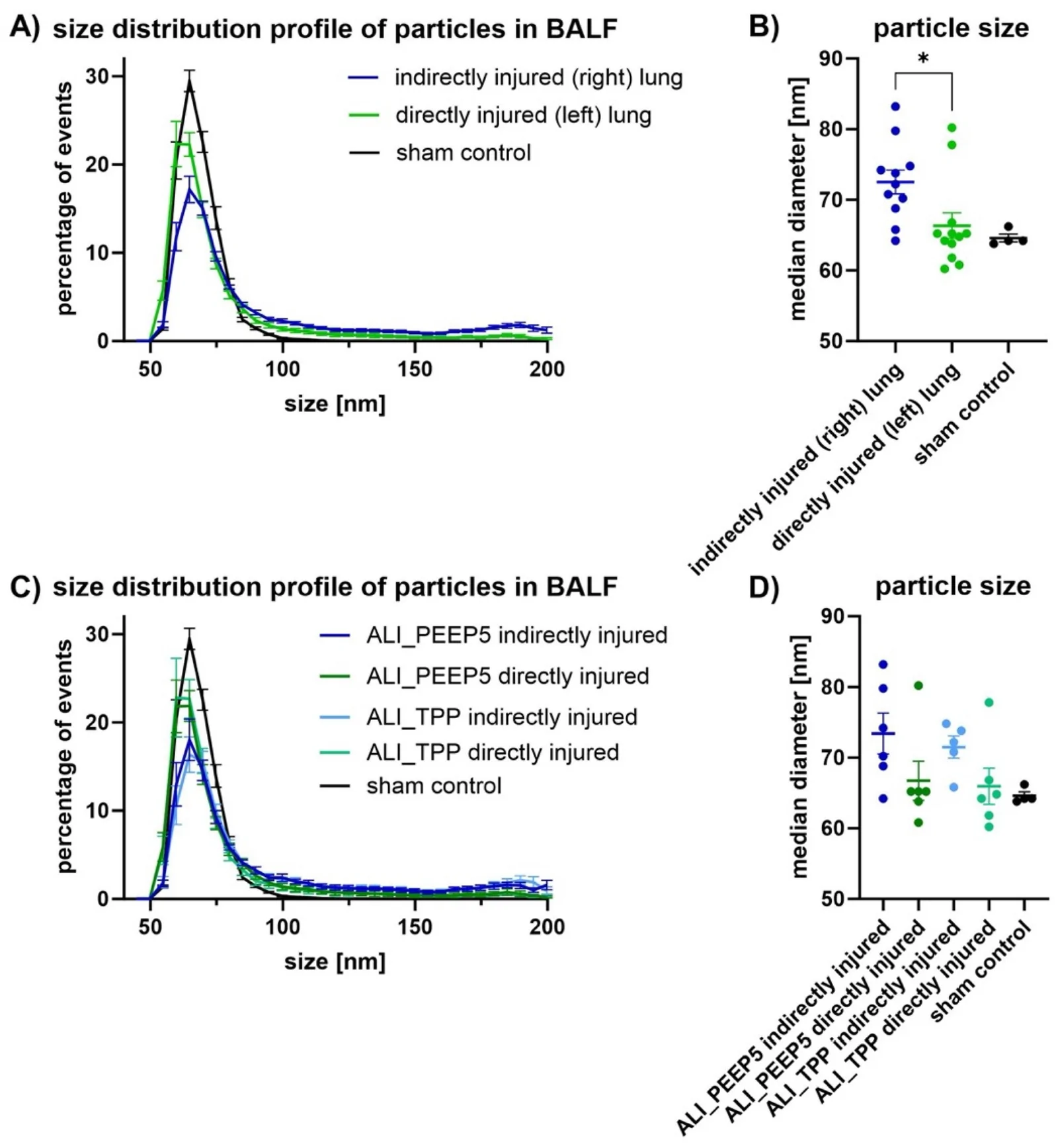
**Size distribution and median particle size of BALF-derived EVs.** (**A**,**C**) Extracellular vesicle (EV) size distribution profiles obtained from bronchoalveolar lavage fluid (BALF) of the indirectly injured contralateral (right) lung, the directly injured (left) lung, and sham controls. Panels (**A**,**C**) display the percentage of detected events across particle diameters, measured by nano-flow cytometry (NanoAnalyzer). Panel (**C**) includes the comparison of fixed positive end-expiratory pressure (PEEP; 5 cmH_2_O) versus individualized PEEP targeting a transpulmonary pressure at end-expiration of 0–3 cmH_2_O. (**B**,**D**) Median EV particle size derived from the same lung regions and ventilation strategies. Data represent biological replicates from individual lungs, displayed as individual data points with line at mean ± SEM. Sample numbers may differ because predefined outliers were excluded. Statistical analysis was performed using one-way ANOVA with Tukey’s post hoc test; significance is indicated by asterisks (*). Blue = indirectly injured lung; green = directly injured lung; black = sham.

Our methodology enabled precise NanoFCM-based measurement of particle diameters ranging from 40 to 200 nanometers, providing a comprehensive overview of the EV size spectrum measured in BALF. To describe the particle population, size distribution profiles are presented as percentage-of-events histograms (Figure 3A). Across all groups, size distribution profiles showed a dominant mode close to the reported median, indicating that the median size represents the central EV population.

Following the analytical approach used for EV concentration analysis (Section 2.1.2), particle size profiles were first analyzed independent of ventilation strategy. Median particle size differed significantly among directly injured, indirectly injured, and sham lungs (*p* = 0.0183). Median EV size was significantly smaller in directly injured compared with indirectly injured lungs (66.33 ± 1.80 nm vs. 72.53 ± 1.69 nm; *p* = 0.035; Figure 3B), while sham lungs showed sizes comparable to directly injured lungs (64.60 ± 0.54 nm vs. 66.33 ± 1.80 nm; *p* = 0.853).

In a second step, stratification by ventilation strategy revealed no significant differences in EV size distribution or median size between ALI_PEEP5, ALI_TPP, and sham lungs in either lung region (all *p* > 0.091; Figure 3C,D).

### 2.2. Histologic Findings

**Figure 4:**
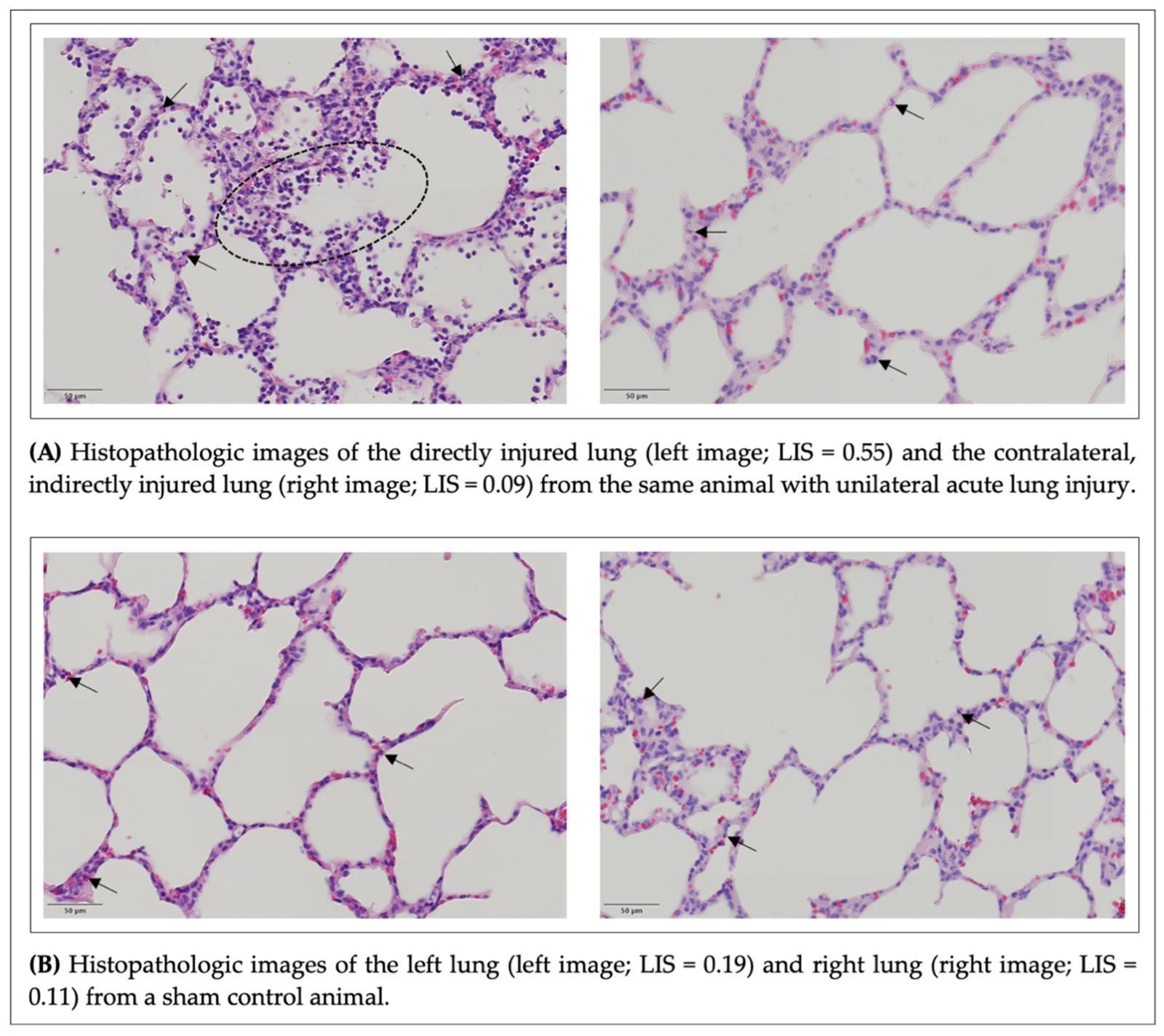
**Histopathologic characterization of lung injury.** Representative hematoxylin and eosin–stained lung sections at 400× magnification from (**A**) an injured lung system and (**B**) a sham control. In panel (**A**), the left image shows the directly injured lung and the right image the contralateral, indirectly injured lung from the same animal with unilateral acute lung injury (ALI). Panel (**B**) presents representative sections of the left (left image) and right (right image) lungs from a sham animal. Corresponding lung injury scores (LIS) are indicated. Scale bar = 50 µm. The directly injured lung (Panel (**A**), left image) displays characteristic features of early ALI, including intra-alveolar neutrophil infiltration (example delineated by dotted lines), eosinophilic intra-alveolar edema, and interstitial neutrophil accumulation (arrows). In contrast, the indirectly injured lung (Panel (**A**), right image) shows only mild interstitial neutrophil infiltration (arrows) and preserved alveolar architecture without intra-alveolar neutrophils, similar to the sham lungs (Panel (**B**)).

As previously described in detail [12], histology confirmed the marked asymmetry of ALI in the experimental model. The principal histologic findings are summarized here to provide the necessary context for the present secondary analysis. Directly injured lungs exhibited extensive intra-alveolar edema, alveolar and interstitial neutrophilic infiltration, and disruption of the alveolar architecture, whereas contralateral lungs showed only mild interstitial alterations with preserved alveolar structure, comparable to sham lungs (Figure 4). Accordingly, LIS differed significantly among directly injured, indirectly injured, and sham lungs, with higher scores in directly injured lungs than in indirectly injured and sham lungs. No significant differences were observed between indirectly injured and sham lungs, consistent with the finding that histologic injury remained largely confined to the lavaged lung throughout the six-hour observation period despite the presence of secondary mechanical stress in the initially non-injured contralateral lung [13]. Stratification by ventilation strategy revealed no significant differences between the ALI_PEEP5 and ALI_TPP groups in either lung region.

### 2.3. Correlation of EV Metrics with Histologic Injury

Correlations between BALF-derived EV metrics (median particle size and concentration) and histologic injury (LIS, alveolar, and interstitial neutrophils) were analyzed using pooled samples across ventilation strategies.

#### 2.3.1. Median EV Size

**Figure 5:**
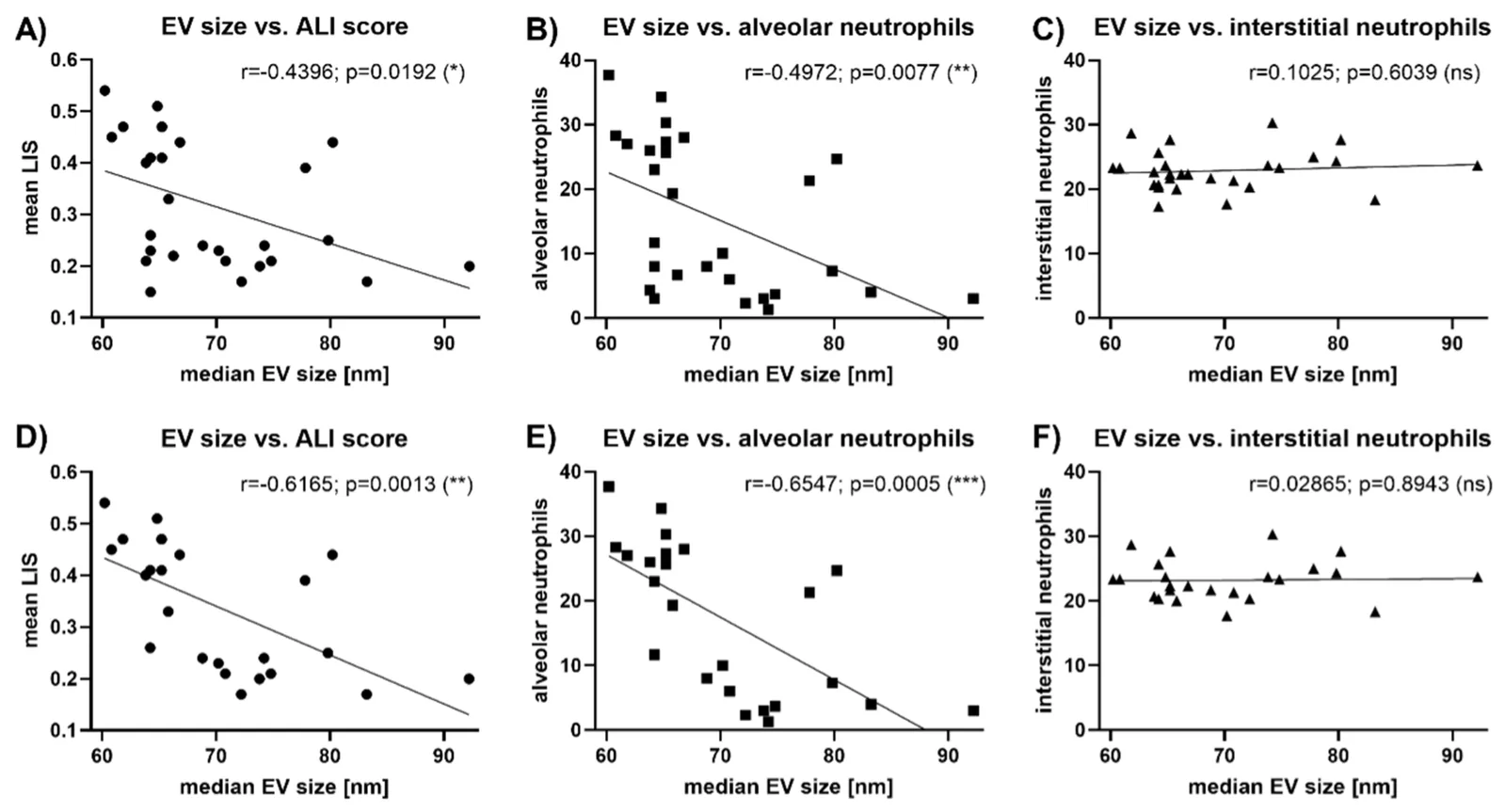
**Correlation of BALF-derived EV size with histologic lung injury parameters.** (**A**–**F**) Pearson correlations between median extracellular vesicle (EV) particle size in bronchoalveolar lavage fluid (BALF) and histologic indicators of lung injury in the porcine acute lung injury model. Subpanels display correlations with (**A**,**D**) the modified lung injury score (LIS), (**B**,**E**) mean alveolar neutrophil counts, and (**C**,**F**) interstitial neutrophil counts. Panels (**A**–**C**) show all samples across experimental groups, whereas panels (**D**–**F**) show the corresponding analyses after exclusion of sham animals. Each data point represents one biological replicate. Data represent biological replicates from individual lungs. Sample numbers may differ because predefined outliers were excluded. Simple linear regression lines are shown; Pearson’s *r* and corresponding *p*-values are provided within each panel; ns = not significant, * *p* < 0.05, ** *p* < 0.01, *** *p* < 0.001.

Median EV size showed significant inverse correlations with histologic injury: smaller EVs were associated with higher LIS (Pearson *r* = −0.44, *p* = 0.019; Figure 5A) and greater neutrophil counts in the alveolar space (Pearson *r* = −0.50, *p* = 0.008; Figure 5B). No significant correlation was observed with interstitial neutrophil counts (Pearson *r* = 0.10, *p* = 0.604, Figure 5C).

We performed a follow-up analysis excluding sham animals to avoid floor effects associated with their minimal lung injury and to examine correlations within the dynamic range of injury severity. Removing sham animals reduces variance compression at the lower end of the injury scale and enables a more accurate assessment of EV–injury relationships across injured lungs only. When excluding shams, the inverse relationships between median EV size, LIS, and alveolar neutrophils remained significant and became even more pronounced (LIS: Pearson *r* = –0.62, *p* = 0.001; alveolar neutrophils: Pearson *r* = –0.65, *p* = 0.001; Figure 5D,E). In contrast, no correlation was observed between EV size and interstitial neutrophils (Pearson *r* = 0.03, *p* = 0.89; Figure 5F).

#### 2.3.2. EV Concentration

EV concentration did not correlate significantly with LIS (Pearson *r* = 0.16, *p* = 0.421 and *r* = −0.06, *p* = 0.794; Figure S1A,D), alveolar neutrophils (Pearson *r* = 0.11, *p* = 0.583 and *r* = −0.11, *p* = 0.634; Figure S1B,E), or interstitial neutrophils (Pearson *r* = 0.29, *p* = 0.155 and *r* = 0.22, *p* = 0.321; Figure S1C,F), even when sham animals were excluded from the analysis.

#### 2.3.3. Exploratory Integrative Analyses

**Figure 6:**
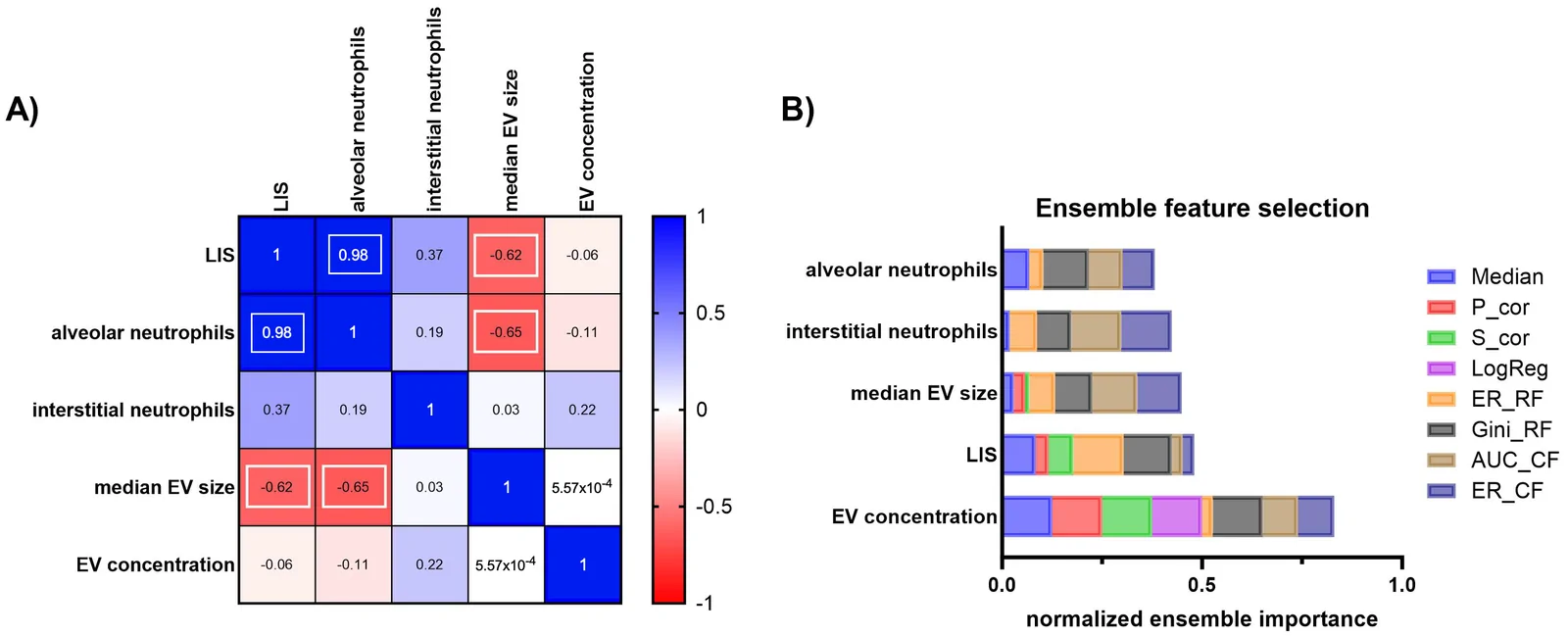
**Exploratory integrative analyses of EV-injury associations**. (**A**) Pearson correlation matrix including all extracellular vesicles (EVs) and histologic parameters across experimental groups. The heatmap displays Pearson correlation coefficients (*r*) according to the color scale (blue = positive correlation, red = negative correlation), and statistically significant correlations are highlighted by white squares. (**B**) Ensemble feature selection analysis of all EV and histologic parameters. The cumulative bar plot summarizes the relative contribution of individual variables across the integrated feature selection methods. The selected feature is EV concentration. Abbreviations: P_cor, Pearson product–moment correlation; LogReg, logistic regression; ER_RF, error-rate-based variable importance measure from random forest; Gini_RF, Gini-index-based variable importance measure from randomForest; AUC_CF, area under the curve (cforest); ER_CF, error-rate-based variable importance measure (cforest).

A correlation heatmap summarizing pairwise Pearson coefficients among EV metrics and histologic variables is provided in Figure 6A to visualize the overall pattern of associations. In addition, EFS identified EV concentration as the top discriminator between directly injured and contralateral lungs (normalized ensemble importance = 0.83), supporting its potential as a marker of pulmonary stress (Figure 6B).

**Figure 7:**
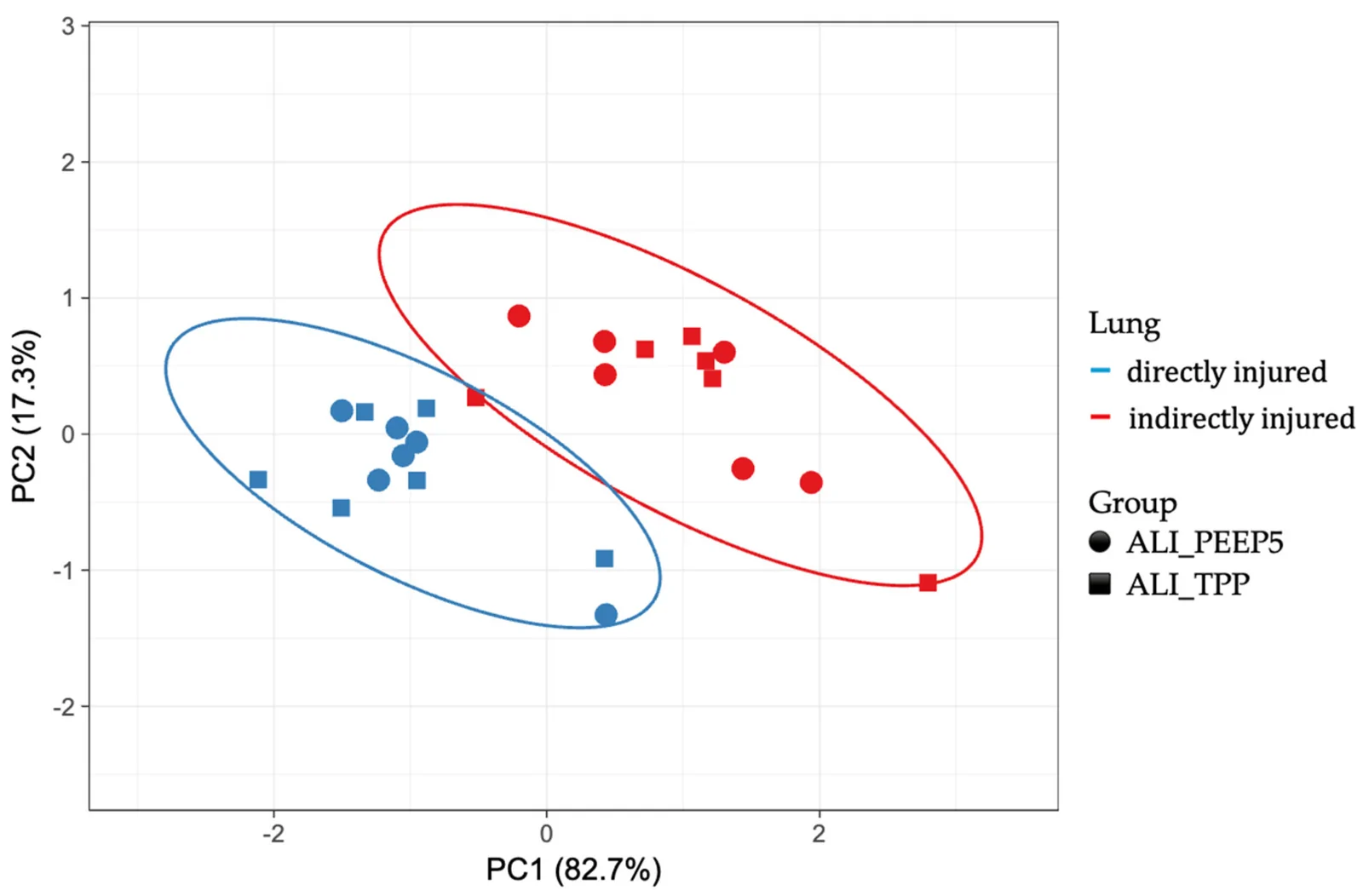
**Principal component analysis of BALF-derived EV size and alveolar neutrophils.** Principal component analysis (PCA) of bronchoalveolar lavage fluid (BALF)-derived extracellular vesicle (EV) median particle size and alveolar neutrophil counts. Each point represents one biological replicate. Red symbols indicate contralateral (indirectly injured) lungs, and blue symbols indicate directly injured lungs. Circles denote animals ventilated with fixed low positive end-expiratory pressure (PEEP; 5 cmH_2_O), and squares denote animals ventilated with individualized PEEP targeting a transpulmonary pressure at end-expiration of 0–3 cmH_2_O. Axes display the first two principal components. Data represent biological replicates from individual lungs. Sample numbers may differ because predefined outliers were excluded.

In line with these findings, principal component analysis (PCA) based on EV size and alveolar neutrophil counts demonstrated clear separation between directly injured and contralateral indirectly injured lungs, whereas no distinct clustering was observed between different PEEP strategies (Figure 7).

## 3. Discussion

In this translational porcine model of unilateral ALI, we show that NanoFCM-based profiling of BALF-derived EVs captures distinct aspects of early lung injury. While EV concentration differentiated injured from non-injured lungs irrespective of the underlying injury pattern, median EV size distinguished direct inflammatory injury from secondary mechanical stress and correlated closely with histologic injury severity and alveolar neutrophil infiltration. To strengthen the methodological robustness of these findings, the vesicular identity of BALF-derived particles was confirmed using complementary TEM and tetraspanin dot blot analyses in accordance with current MISEV recommendations [2]. These orthogonal approaches demonstrated the characteristic morphology of EVs together with the presence of EV-associated markers, supporting the interpretation that the particles analyzed by NanoFCM predominantly represent EVs.

Reliable biomarkers for the early detection of localized molecular alterations before clinically overt ARDS remain limited. Because BALF directly reflects the alveolar compartment, BALF-derived EVs may provide a spatially resolved molecular readout of pulmonary injury and inflammatory activity at the site of injury [8,9,14]. In contrast to circulating EVs, which integrate signals from multiple organs, BALF-derived EVs enable characterization of regional pulmonary responses and may therefore detect regional injury processes before they become systemically apparent [15,16]. The translational relevance of this approach is further supported by the molecular similarity between porcine and human BALF [17]. Moreover, the NanoFCM workflow applied in this study permits direct quantification of EVs in native BALF without prior isolation, thereby minimizing isolation-related bias and selective enrichment of specific vesicle populations while requiring only small BALF volumes for analysis [18,19].

Previous experimental and clinical studies have consistently demonstrated increased EV concentrations in BALF during inflammatory lung injury, supporting the concept that EV release represents a general response to pulmonary stress [20,21,22,23,24,25]. In line with these findings, BALF-derived EV concentrations were significantly increased in both directly injured and contralateral mechanically stressed lungs compared with sham controls, despite marked differences in histologic injury severity. This observation suggests that increased EV release is not restricted to sites of overt tissue damage but may also reflect early biological responses to secondary mechanical loading of lung areas during early asymmetric injury. Accordingly, our findings support the concept that EV concentration reflects the presence of pulmonary stress rather than the specific mechanism of injury. While EV quantification alone may therefore serve as a sensitive indicator of early lung injury, it appears insufficient for distinguishing between distinct underlying injury processes.

In contrast to EV concentration, median EV size differed significantly between lung sides despite similarly increased EV concentrations in both lungs. Smaller EVs, predominantly found in the directly injured lung, were consistently associated with greater histologic injury severity and increased alveolar neutrophil infiltration, suggesting that EV size may provide additional information beyond the presence of pulmonary stress alone. Previous studies have similarly demonstrated that EV characteristics vary according to the underlying pathophysiological stimulus, indicating that distinct forms of lung injury may be associated with different EV populations [21,22,24,25]. Together with our findings, these observations support the concept that EV size may reflect the biological nature of the underlying injury rather than merely its presence.

A biologically plausible explanation for these observations may be a shift in the predominant cellular origin of BALF-derived EVs [26]. Previous studies have shown that both inflammatory cells, including neutrophils, and structural lung cells, such as alveolar epithelial and endothelial cells, release extracellular vesicles in response to different forms of lung injury and mechanical stress [21,24,25,27,28]. Therefore, it is conceivable that the differences in EV size observed in our study may reflect shifts in the relative contribution of these cellular sources rather than changes in overall EV release alone. However, the cellular origin of BALF-derived EVs could not be determined in the present study because validated porcine cell-specific EV markers are currently lacking. Therefore, our observations should be regarded as hypothesis-generating and require future validation using approaches capable of directly identifying the cellular origin of BALF-derived EVs. Taken together, our findings support the concept that EV size may complement EV concentration by providing additional information on the nature and severity of early lung injury.

Beyond their potential diagnostic value, BALF-derived EVs may also provide a sensitive molecular readout for evaluating the biological effects of lung-protective ventilation strategies. Previous work in a comparable unilateral ALI model demonstrated that a TPP-guided PEEP strategy may reduce regional mechanical stress by promoting a more balanced distribution of tidal volume between injured and non-injured lung regions [13]. Whether these mechanical benefits are accompanied by attenuation of local biotrauma, however, has not yet been fully elucidated. In the present study, BALF-derived EV concentrations were consistently lower in both directly and indirectly injured lungs in animals with a TPP-guided PEEP strategy compared with fixed low PEEP. Although these differences did not reach statistical significance in this exploratory analysis, the consistent directional trend across both lungs is noteworthy. This observation is supported by previous clinical and experimental studies demonstrating that elevated BALF-derived EV concentrations are associated with increased disease severity and adverse outcomes in ALI, whereas declining EV concentrations over time have been linked to clinical recovery [10,20,29]. Together with these findings, our observations raise the possibility that BALF-derived EVs may provide a molecular readout not only for the early detection and characterization of lung injury but also for evaluating biological responses to individualized ventilatory interventions. However, this interpretation remains hypothesis-generating and requires confirmation in adequately powered studies specifically designed to evaluate ventilation-induced changes in EV release.

Several limitations should be considered when interpreting our findings. First, although complementary TEM and tetraspanin dot blot analyses substantially strengthen the identification of BALF-derived particles as EVs, NanoFCM-based particle analysis alone cannot completely exclude co-detection of similarly sized non-vesicular extracellular particles. While this is unlikely to substantially confound our sterile experimental ALI model, it should be considered when translating this approach to clinical BALF samples, particularly in infectious lung injury [30,31]. Second, the absence of validated porcine cell-specific EV markers precluded direct determination of the cellular origin of BALF-derived EVs. Consequently, the proposed shift in the relative contribution of inflammatory and structural cell-derived EV populations should be regarded as hypothesis-generating and requires confirmation in future mechanistic studies. Third, our analysis was intentionally limited to basic physical EV characteristics, namely concentration and size, rather than molecular EV characteristics, including cargo composition. Consequently, the molecular mechanisms underlying the observed changes in EV profiles remain unresolved. Furthermore, measurements were performed at a single early time point following ALI induction. Consequently, temporal changes in EV release and their relationship to disease progression or resolution could not be assessed. Finally, the inherent limitations of a controlled porcine unilateral ALI model apply. Species-specific differences, controlled ventilation under general anesthesia, and the experimental nature of the injury limit direct extrapolation to the heterogeneous clinical spectrum of ARDS. In addition, ethical considerations inherent to large-animal research limited sample size. Consequently, several analyses—including the exploratory comparison of ventilatory strategies—should be interpreted cautiously and confirmed in adequately powered future studies.

## 4. Materials and Methods

### 4.1. Study Design and Ethical Approval

This study represents a predefined secondary analysis of a prospective, randomized, controlled porcine experiment [12]. Histologic data have been reported previously and are referenced here for correlation with EV metrics. Seventeen healthy domestic male and female pigs (German Landrace; 4–5 months of age, 50 ± 6 kg) were anesthetized, surgically instrumented, mechanically ventilated, and randomized (coin toss) to two experimental groups receiving either a fixed PEEP of 5 cmH_2_O (ALI_PEEP5) or TPP-guided PEEP titration targeting a TPP_exp_ of 0–3 cmH_2_O (ALI_TPP), or to a sham group. Unilateral ALI was subsequently induced by Triton^®^ X-100 (Merck KGaA, Darmstadt, Germany) lavage of the left lung in the animals assigned to the experimental groups, whereas sham animals underwent bronchoscopy without lavage. Following ALI induction, three animals developed a pneumothorax and were excluded because this condition could substantially alter lung mechanics. Consequently, the final study population comprised fourteen animals: six in the ALI_PEEP5 group, six in the ALI_TPP group, and two sham animals. Sham animals were mechanically ventilated using a fixed PEEP of 5 cmH_2_O, identical to the ALI_PEEP5 group, but did not undergo ALI induction. Histologic and EV analyses were performed by investigators blinded to group allocation. All procedures were approved by the responsible animal ethics authority (Regierungspräsidium Karlsruhe, Karlsruhe-Innenstadt-West, Germany, No. G-123/23, approved on 4 September 2023) and conducted in accordance with applicable regulations. The animals were housed at the interfaculty biomedical facility of the University of Heidelberg, Germany, and sourced from a local pig breeder.

EV procedures and reporting followed the MISEV2023 guidelines [2]. EVs were defined as lipid bilayer-enclosed particles <200 nm in BALF. EV concentration and size were determined by NanoFCM, while complementary transmission electron microscopy and tetraspanin dot blot analyses were performed to support EV characterization. Direct attribution of EVs to specific cellular origins remained beyond the scope of the present study due to the absence of validated porcine cell-specific EV markers.

Potential confounding was minimized by standardized experimental procedures, randomization, and blinded histologic and EV analyses. Group allocation was known to the investigators conducting the animal experiments.

### 4.2. Animal Preparation and ALI Induction

Anesthesia, surgical instrumentation, and animal preparation followed previously published protocols [12]. Independent lung ventilation was achieved using a modified double-lumen tube following tracheotomy [32]. An esophageal balloon catheter was placed for esophageal pressure measurement as a surrogate for pleural pressure [13].

In experimental groups, unilateral ALI was induced in the left lung by bronchoscopic lavage with 0.3% Triton X-100, as previously described [12]. Sham animals underwent bronchoscopy without lavage. Because sham animals did not undergo unilateral ALI induction, both lungs served as non-injured control samples throughout the experiment.

### 4.3. Experimental Protocol

Following ALI induction or sham bronchoscopy, all animals were ventilated using volume-controlled ventilation (tidal volume 6 mL/kg). PEEP was applied according to group allocation: a fixed PEEP of 5 cmH_2_O (sham, n = 2; ALI_PEEP5, n = 6) or individualized PEEP adjusted hourly to maintain a TPP_exp_ between 0 and 3 cmH_2_O (ALI_TPP, n = 6) as previously described [12]. After six hours, BALF was collected separately from the left and right lungs of all animals, followed by euthanasia and lung tissue sampling.

### 4.4. BALF Collection and Processing

BALF was obtained by bronchoscopy using a 20 mL aliquot of sterile isotonic saline per lung. Samples were immediately centrifuged (5 min at 300× *g*, 4 °C; 10 min at 2000× *g*, 4 °C), followed by subsequent centrifugation of the supernatant (30 min at 10,000× *g*, 4 °C). The final supernatant was stored at −80 °C until further processing.

### 4.5. EV Quantification and Size Analysis

Particle concentration and size distribution in BALF were determined by nano-flow cytometry (NanoAnalyzer, NanoFCM Co., Ltd., Nottingham, UK). Measurements were performed by an investigator blinded to group allocation. The instrument was calibrated for particle concentration and size using manufacturer-provided reference beads and silica nanoparticle standards. BALF samples were diluted to achieve optimal event rates, and data were acquired under continuous flow. Median particle size and concentration were used for statistical analyses.

### 4.6. EV Isolation from BALF

To enable orthogonal characterization of EVs in accordance with current MISEV recommendations, representative BALF samples were subjected to EV enrichment for confirmatory TEM and dot blot analyses [2]. BALF samples from four animals per experimental group were pooled (50 µL per sample; total volume 200 µL). Sham samples were pooled irrespective of lung side, whereas BALF obtained from the directly injured (left) and contralateral mechanically stressed (right) lungs of both ALI groups was processed separately.

Pooled BALF samples were centrifuged at 10,000× *g* for 20 min at 4 °C to remove residual cellular debris and larger particles. Supernatants were transferred to ultracentrifuge tubes, adjusted to equal volumes using 0.1 µm-filtered phosphate-buffered saline (PBS; Cytiva, Marlborough, MA, USA), and ultracentrifuged at 100,000× *g* for 90 min at 4 °C using a Sorvall™ Discovery™ 90SE ultracentrifuge equipped with an F50L-24×1.5 fixed angle rotor (Thermo Fisher Scientific, Waltham, MA, USA). Following ultracentrifugation, the supernatant was discarded, and EV pellets were resuspended in 30 µL of 0.1 µm-filtered PBS (Cytiva Hyclone, South Logan, UT, USA) for subsequent TEM and dot blot analyses.

### 4.7. Dot Blot Analysis

Dot blot analysis was performed to confirm the presence of EV-associated tetraspanins in isolated BALF-derived EV preparations. Five microliters of each EV suspension were spotted onto nitrocellulose membranes and allowed to air dry completely. Membranes were blocked for 1 h at room temperature in Tris-buffered saline containing 0.1% Tween-20 (TBS-T) supplemented with 10% (*w*/*v*) skim milk and 5% bovine serum albumin (BSA).

Following three washing steps (5 min each) with TBS-T, membranes were incubated for 2 h at room temperature with a tetraspanin antibody cocktail consisting of anti-CD9 (BD Biosciences, Franklin Lakes, NJ, USA; #555370), anti-CD63 (Santa Cruz Biotechnology, Dallas, TX, USA; sc-5275), and anti-CD81 (Santa Cruz Biotechnology; sc-7631), each diluted 1:500 in TBS-T containing 3% BSA (blocking buffer).

Membranes were subsequently washed three times for 5 min with TBS-T and incubated for 1 h at room temperature with HRP-conjugated mouse IgG kappa binding protein (m-IgGκ BP; Santa Cruz Biotechnology, Dallas, TX, USA; sc-516102) diluted 1:2000 in blocking buffer. After three additional washing steps with TBS-T, immunoreactive signals were visualized using enhanced chemiluminescence detection reagent and recorded with an imaging system (Intas Science Imaging Instruments, Göttingen, Germany).

### 4.8. Transmission Electron Microscopy

Transmission electron microscopy was performed to confirm the presence and morphology of EVs. Carbon-coated 400-mesh copper grids were rendered hydrophilic by glow discharge (PELCO easiGlow, Ted Pella, Redding, CA, USA). Subsequently, 5 µL of each EV suspension were applied to the grids, briefly washed with double-distilled water, and negatively stained with 2% (*w*/*v*) uranyl acetate. Samples were examined using a JEOL JEM-2100 transmission electron microscope (JEOL Ltd., Tokyo, Japan) operated at an accelerating voltage of 120 kV. Digital images were acquired using an F214 FastScan CCD camera (TVIPS, Gauting, Germany).

### 4.9. Histologic Assessment

Histologic evaluation followed previously published methods [12]. Lung tissue sections were stained with hematoxylin and eosin and assessed by two independent, blinded pathologists. Lung injury was quantified using a refined lung injury score incorporating alveolar and interstitial neutrophil infiltration and alveolar septal thickening, as described previously [12]. LIS values were used for comparison with EV metrics.

### 4.10. Ensembled Feature Selection

Ensemble feature selection (EFS) was used as an exploratory approach to identify variables contributing to group separation, integrating multiple feature selection algorithms into a single importance score to reduce method-specific bias [33,34]. EV-related and histologic parameters were included, and features were ranked according to their relative contribution to group discrimination.

### 4.11. Statistical Analysis

The sample size was determined for the original experimental study based on expected effect sizes derived from previous studies and pilot experiments. As the present work represents a secondary analysis of BALF samples collected within that study, no separate a priori sample size calculation was performed for the extracellular vesicle analyses.

Statistical analyses and graphical visualization were performed using SPSS (Version 27.0, IBM Corp., Armonk, NY, USA) and GraphPad Prism (Version 10.3.1, GraphPad Software, Inc., La Jolla, CA, USA). Data are presented as mean ± SEM, and *p* < 0.05 was considered statistically significant. Histopathologic comparisons were performed using one-way ANOVA with Bonferroni post hoc testing. EV metrics were analyzed using one-way ANOVA with Tukey’s post hoc test. Pearson correlation analyses were conducted to examine associations between EV metrics and histologic injury parameters; for this purpose, all samples were pooled across ventilation strategies. Additionally, graphs present regression lines calculated by simple linear regression.

Where applicable, sample numbers differ because two BALF samples from indirectly injured lungs were excluded following predefined outlier detection.

TEM and dot blot analyses were performed as qualitative confirmatory experiments for EV characterization and were therefore not subjected to statistical analysis.

## 5. Conclusions

In this porcine model of early asymmetric ALI, BALF-derived EV profiling provided complementary molecular information on early lung injury. Whereas increased EV concentration was associated with overall pulmonary stress, differences in EV size differentiated direct inflammatory injury from secondary mechanical stress and correlated closely with histologic injury severity, particularly the extent of alveolar neutrophil infiltration. Furthermore, exploratory analyses suggested that BALF-derived EV profiling may also provide a molecular readout for evaluating the biological response to individualized ventilatory strategies. Importantly, these insights were obtained using basic and readily accessible EV metrics. Taken together, our findings support the concept that quantitative and size-based BALF-derived EV profiling may contribute to the early detection, characterization, and biological monitoring of acute lung injury. Further validation in translational and clinical studies is warranted.

## Data Availability

The data that support the findings of this study are available from the corresponding author upon reasonable request.

## Supplementary Materials

The following supporting information can be downloaded at https://www.mdpi.com/article/10.3390/ijms27167173/s1.

## Abbreviations

The following abbreviations are used in this manuscript:

ALI	Acute lung injury
ALI_PEEP5	ALI group with fixed PEEP of 5 cmH_2_O
ALI_TPP	ALI group with transpulmonary-pressure guided PEEP
ANOVA	Analysis of variance
APC	Article processing charge
ARDS	Acute respiratory distress syndrome
AUC	Area under the curve
BALF	Bronchoalveolar lavage fluid
BSA	bovine serum albumin
EFS	Ensemble feature selection
EVs	Extracellular vesicles
LIS	Lung injury score
MISEV2023	Minimal information for studies of EVs
NanoFCM	nano-flow cytometry
PCA	Principal component analysis
PEEP	Positive end-expiratory pressure
SEM	Standard error of the mean
TBS-T	Tris-buffered saline containing 0.1% Tween-20
TEM	transmission electron microscopy
TPP	Transpulmonary pressure
TPP_exp_	TPP at end-expiration
VILI	Ventilator-induced lung injury

## References

[1] Yáñez-Mó M., Siljander P.R.-M., Andreu Z., Bedina Zavec A., Borràs F.E., Buzas E.I., Buzas K., Casal E., Cappello F., Carvalho J. (2015). Biological properties of extracellular vesicles and their physiological functions. J. Extracell. Vesicles.

[2] Welsh J.A., Goberdhan D.C.I., O’DRiscoll L., Buzas E.I., Blenkiron C., Bussolati B., Cai H., Di Vizio D., Driedonks T.A.P., Erdbrügger U. (2024). Minimal information for studies of extracellular vesicles (MISEV2023): From basic to advanced approaches. J. Extracell. Vesicles.

[3] Bellani G., Laffey J.G., Pham T., Fan E., Brochard L., Esteban A., Gattinoni L., Van Haren F., Larsson A., McAuley D.F. (2016). Epidemiology, Patterns of Care, and Mortality for Patients With Acute Respiratory Distress Syndrome in Intensive Care Units in 50 Countries. JAMA.

[4] Matthay M.A., Arabi Y., Arroliga A.C., Bernard G., Bersten A.D., Brochard L.J., Calfee C.S., Combes A., Daniel B.M., Ferguson N.D. (2024). A New Global Definition of Acute Respiratory Distress Syndrome. Am. J. Respir. Crit. Care Med..

[5] Costa A., Sakho B., Gomez S., Khanyan B., Leybengrub P., Bergese S. (2025). Ventilator-Associated Lung Injury: Pathophysiology, Prevention, and Emerging Therapeutic Strategies. Int. J. Mol. Sci..

[6] Pham T., Pesenti A., Bellani G., Rubenfeld G., Fan E., Bugedo G., Lorente J.A., Fernandes A.D.V., Van Haren F., Bruhn A. (2021). Outcome of acute hypoxaemic respiratory failure: Insights from the LUNG SAFE Study. Eur. Respir. J..

[7] Gattinoni L., Marini J.J. (2022). In search of the Holy Grail: Identifying the best PEEP in ventilated patients. Intensive Care Med..

[8] Raeven P., Zipperle J., Drechsler S. (2018). Extracellular Vesicles as Markers and Mediators in Sepsis. Theranostics.

[9] Mohan A., Agarwal S., Clauss M., Britt N.S., Dhillon N.K. (2020). Extracellular vesicles: Novel communicators in lung diseases. Respir. Res..

[10] Park K.S., Lasser C., Lotvall J. (2025). Extracellular vesicles and the lung: From disease pathogenesis to biomarkers and treatments. Physiol. Rev..

[11] Li P., Wu Y., Goodwin A.J., Wolf B., Halushka P.V., Wang H., Zingarelli B., Fan H. (2023). Circulating extracellular vesicles are associated with the clinical outcomes of sepsis. Front. Immunol..

[12] Mutschler C.H., Seybold B., Aschauer S., Englert N., Weis C.-A., Poth T., Cetiner D., Wielpütz M.O., Kehr D., Weigand M.A. (2025). Optimizing Positive End-Expiratory Pressure in Asymmetric Acute Lung Injury in a Porcine Model: The Role of Transpulmonary Pressure. Int. J. Mol. Sci..

[13] Bastia L., Engelberts D., Osada K., Katira B.H., Damiani L.F., Yoshida T., Chen L., Ferguson N.D., Amato M.B.P., Post M. (2021). Role of Positive End-Expiratory Pressure and Regional Transpulmonary Pressure in Asymmetrical Lung Injury. Am. J. Respir. Crit. Care Med..

[14] Davidson K.R., Ha D.M., Schwarz M.I., Chan E.D. (2020). Bronchoalveolar lavage as a diagnostic procedure: A review of known cellular and molecular findings in various lung diseases. J. Thorac. Dis..

[15] Soni S., Wilson M.R., O’DEa K.P., Yoshida M., Katbeh U., Woods S.J., Takata M. (2016). Alveolar macrophage-derived microvesicles mediate acute lung injury. Thorax.

[16] Zareba L., Szymanski J., Homoncik Z., Czystowska-Kuzmicz M. (2021). EVs from BALF-Mediators of Inflammation and Potential Biomarkers in Lung Diseases. Int. J. Mol. Sci..

[17] Mutschler D.K., Larsson A.O., Basu S., Nordgren A., Eriksson M.B. (2002). Effects of mechanical ventilation on platelet microparticles in bronchoalveolar lavage fluid. Thromb. Res..

[18] Baranyai T., Herczeg K., Onódi Z., Voszka I., Módos K., Marton N., Nagy G., Mäger I., Wood M.J., El Andaloussi S. (2015). Isolation of Exosomes from Blood Plasma: Qualitative and Quantitative Comparison of Ultracentrifugation and Size Exclusion Chromatography Methods. PLoS ONE.

[19] Lee N., Kim D., Cho H., Jeong G., Lee S.J., Lee H. (2024). Protocol to isolate and characterize pulmonary-specific extracellular vesicles in mice. STAR Protoc..

[20] Bastarache J.A., Fremont R.D., Kropski J.A., Bossert F.R., Ware L.B. (2009). Procoagulant alveolar microparticles in the lungs of patients with acute respiratory distress syndrome. Am. J. Physiol. Lung Cell Mol. Physiol..

[21] Moon H.G., Cao Y., Yang J., Lee J.H., Choi H.S., Jin Y. (2015). Lung epithelial cell-derived extracellular vesicles activate macrophage-mediated inflammatory responses via ROCK1 pathway. Cell Death Dis..

[22] Lee H., Zhang D., Laskin D.L., Jin Y. (2018). Functional Evidence of Pulmonary Extracellular Vesicles in Infectious and Noninfectious Lung Inflammation. J. Immunol..

[23] Yuan Z., Bedi B., Sadikot R.T. (2018). Bronchoalveolar Lavage Exosomes in Lipopolysaccharide-induced Septic Lung Injury. J. Vis. Exp..

[24] Zhang S., Dai H., Zhu L., Lin F., Hu Z., Jing R., Zhang W., Zhao C., Hong X., Zhong J.-H. (2018). Microvesicles packaging IL-1beta and TNF-alpha enhance lung inflammatory response to mechanical ventilation in part by induction of cofilin signaling. Int. Immunopharmacol..

[25] Nirujogi T.S., Kotha S.R., Chung S., Reader B.F., Yenigalla A., Zhang L., Shapiro J.P., Wisler J., Christman J.W., Maddipati K. (2022). Lipidomic Profiling of Bronchoalveolar Lavage Fluid Extracellular Vesicles Indicates Their Involvement in Lipopolysaccharide-Induced Acute Lung Injury. J. Innate Immun..

[26] Hu Q., Zhang S., Yang Y., Yao J.-Q., Tang W.-F., Lyon C.J., Hu T.Y., Wan M.-H. (2022). Extracellular vesicles in the pathogenesis and treatment of acute lung injury. Mil. Med. Res..

[27] Letsiou E., Alves L.G.T., Felten M., Mitchell T.J., Müller-Redetzky H.C., Dudek S.M., Witzenrath M. (2021). Neutrophil-Derived Extracellular Vesicles Activate Platelets after Pneumolysin Exposure. Cells.

[28] Lee H., Zhang D., Wu J., Otterbein L.E., Jin Y. (2017). Lung Epithelial Cell-Derived Microvesicles Regulate Macrophage Migration via MicroRNA-17/221-Induced Integrin beta(1) Recycling. J. Immunol..

[29] Dong X.-C., Xu X.-Y., Huang Y.-R., Zhu X.-X., Yang Y., Huang W., Liu L. (2023). Prognostic value of secretory autophagosomes in patients with acute respiratory distress syndrome. Biomark. Res..

[30] Noda T. (2011). Native morphology of influenza virions. Front. Microbiol..

[31] Toyofuku M., Nomura N., Eberl L. (2019). Types and origins of bacterial membrane vesicles. Nat. Rev. Microbiol..

[32] Geilen J., Kainz M., Zapletal B., Geleff S., Wisser W., Bohle B., Schweiger T., Schultz M.J., Tschernko E. (2022). Unilateral acute lung injury in pig: A promising animal model. J. Transl. Med..

[33] Neumann U., Genze N., Heider D. (2017). EFS: An ensemble feature selection tool implemented as R-package and web-application. BioData Min..

[34] Neumann U., Riemenschneider M., Sowa J.-P., Baars T., Kälsch J., Canbay A., Heider D. (2016). Compensation of feature selection biases accompanied with improved predictive performance for binary classification by using a novel ensemble feature selection approach. BioData Min..

