# Supplementary material for: Extracellular Vesicles from Bronchoalveolar Lavage Fluid Indicate Early Biomarker Potential and Differentiate Local Lung Injury in a Porcine Model of Asymmetric Acute Lung Injury
Source: Int J Mol Sci. 2026 Aug 11;27(16):7173. doi: 10.3390/ijms27167173 (PMC13513853; doi:10.3390/ijms27167173)
Supplement: Supplementary file 1 [file ijms-27-07173-s001.zip › ijms-4417348-supplementary.pdf]

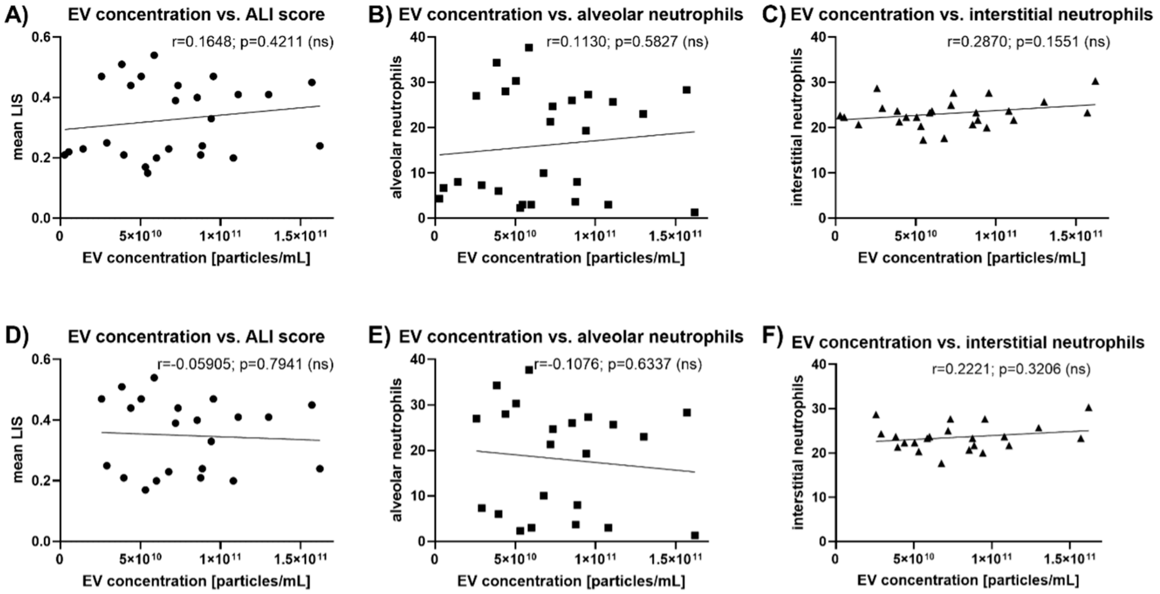


**Figure S1. Correlation of BALF EV concentration with histologic lung injury parameters.** (A–F) Pearson correlations between EV concentration in BALF and histologic indicators of lung injury in the porcine ALI model. Subpanels display correlations with (A+D) the modified lung injury score (LIS), (B+E) mean alveolar neutrophil counts, and (C+F) interstitial neutrophil counts. Panels A-C include all samples across experimental groups, whereas panels D-F present the corresponding analyses after exclusion of sham animals. Each data point represents one biological replicate. Simple linear regression lines are shown; Pearson's *r* and corresponding *p*-values are provided within each panel; ns = not significant.
